# Effectiveness of specialist-delivered interventions in severe mental illness: A systematic review and meta-analysis

**DOI:** 10.1177/00048674251384054

**Published:** 2025-10-22

**Authors:** Tamieka Mawer, Scott Teasdale, Rachel Bacon, Nicholas Brown, Andrew McKune, Jane Kellett

**Affiliations:** 1 University of Canberra, Bruce, ACT, Australia; 2 University of New South Wales, Sydney, NSW, Australia; 3 Mindgardens Neuroscience Network, Sydney, NSW, Australia; 4 Queensland University of Technology, Brisbane, QLD, Australia

**Keywords:** Mental health, nutrition, exercise, health equity

## Abstract

**Objective:**

To establish the effectiveness of specialist-delivered nutrition and exercise interventions on the physical health of people with severe mental illness.

**Methods:**

An electronic database search was completed from earliest record to August 2024 using Scopus, Medline, EMBASE, PsycINFO, and CINAHL, using key nutritional, cardiometabolic and psychiatric terminology. Eligible studies were randomised and non-randomised controlled trials which included specialist-delivered interventions (dietitian and/or nutritionist or exercise professional) with people diagnosed with severe mental illness. Primary outcomes were cardiometabolic risk factors.

**Results:**

Thirty-one studies were included: combined nutrition and exercise intervention (*n* = 12), nutrition intervention only (*n* = 9), and exercise intervention only (*n* = 10), with 23 contributing to the meta-analysis. Meta-analysis of combined nutrition and exercise interventions revealed positive-effects on body mass index (BMI) (Mean diff = −1.78 [95% confidence interval (CI) −2.97 to −0.59], *p* = 0.00) and waist circumference (Mean diff = −4.13 [95% CI −7.25 to −1.00] *p* = 0.01). In nutrition-only intervention studies, the meta-analysis revealed a positive-effect on systolic blood pressure (Mean diff = −6.14 [95% CI −12.02 to −0.26] *p* = 0.04). No significant impacts were tested for exercise-only interventions.

**Conclusion:**

Specialist-delivered nutrition and exercise interventions are effective in improving weight, BMI and waist circumference status over the short to medium term in people diagnosed with severe mental illness. Exercise and nutrition programmes show promising effectiveness, and this research provides evidence to support the implementation as part of standard care of people diagnosed with severe mental illness.

## Introduction

Poor physical health and the association with mental health issues has been well documented (Firth et al., 2019). People who experience psychological health issues are more likely to experience poorer physical health than those who do not have any underlying psychological health issues (Firth et al., 2019). Equally, the likelihood of experiencing mental health issues increase with poor physical health and co-morbidities (Firth et al., 2019). Approximately two in five people will be affected by a mental illness in their lifetime (Khaled et al., 2024; Slade et al., 2025).

Severe mental illness is defined as a mental, behavioural, or emotional disorder resulting in serious functional impairment, which substantially interferes with or limits one or more major life activities (Baron et al., 2022). Schizophrenia and related psychoses, bipolar disorder, and major depression are frequent diagnoses for people experiencing severe mental health challenges (Firth et al., 2019), and they have a two to three times higher average mortality compared to the general population, which translates to a 10- to 20-year reduction in life expectancy (Liu et al., 2017; Walker et al., 2015). Compared with the general population, those who are diagnosed with a severe mental illness are more than two times as likely to develop respiratory disease and/or osteoporosis and higher rates of metabolic syndrome (67% of people managing a severe mental health condition) (Avgerinou et al., 2024; Needham et al., 2025; Ronaldson et al., 2024). Research has also shown the risk of developing diabetes is two to three times more likely in people living with a severe mental illness, and the risk of developing cardiovascular disease is up to four times more likely when compared to the general population (Morgan et al., 2012). Research has found people who are living with a severe mental health condition are more likely to smoke, with the estimates to be as high as 70% of men and 60% of women (Mendelsohn et al., 2015). People experiencing severe mental health challenges are also at an increased risk of being overweight or obese in comparison to the general population (Rajan and Menon, 2017; Stogios et al., 2023). The Royal and Australian and New Zealand College of Psychiatrists (RANZCP) estimates an economic cost of up to $15 billion annually for untreated co-morbidities (Royal Australian & New Zealand College of Psychiatrists, 2016).

Evidence suggests that good nutrition is essential for our mental health and that a number of mental health conditions may be influenced by dietary factors (Grajek et al., 2022). Nutritional problems are more prevalent in people living with mental health issues due to a variety of reasons including reduced access to nutritional food options secondary to low income and high density of fast food restaurants limiting the access to healthier choices (Kent et al., 2022; Mawer et al., 2022), lifestyle behaviours including smoking, alcohol and substance use, and lower levels of physical activity (Bartlem et al., 2015; Dipasquale et al., 2013), poor social determinants including economic instability, limited access to quality health care and access to health screening and health interventions, and the built environment encompassing access to nutritious food, housing and transport (Allen et al., 2014). People with ongoing mental illness may experience lower income, greater social isolation, food insecurity and poverty which are major determinates of health in people with mental illnesses (Kent et al., 2022; Mawer et al., 2022).

Health challenges are further compounded by medication used in the treatment of severe mental health conditions, the negative side effects of antipsychotic medications which include increased appetite, cravings for sugary foods and faster eating (De Hert et al., 2011). Antipsychotics have been shown to be associated with an increased risk of obesity, diabetes mellitus, dyslipidemia, cardiovascular disease and other nutrition-related chronic diseases (Grajek et al., 2022). Second-generation antipsychotics (for example clozapine and olanzapine) are associated with the greatest risk of weight gain (Correll et al., 2015; Dayabandara et al., 2017; Doane et al., 2022), while other antipsychotics (for example, quetiapine, risperidone, and paliperidone) are associated with an intermediate risk of weight gain (Correll et al., 2015; Dayabandara et al., 2017; Doane et al., 2022).

The poor physical health experienced by people managing a severe mental illness has been well documented over recent years (Firth et al., 2019). High rates of physical co-morbidities reduce life expectancy; therefore, the need to include access to a dietitian and exercise specialist as part of standard care has been highlighted (Firth et al., 2019). To date, the effectiveness of specialist-delivered interventions with people living with a severe mental illness including nutrition, exercise and lifestyle interventions, has not been systematically evaluated. The specific questions to be answered by this review are:

a. Do specialist-delivered interventions improve metabolic outcomes (including BMI, weight, waist circumference, heart rate, blood pressure, and biochemistry) of people living with a severe mental illness?
b. Do specialist-delivered interventions improve mental illness symptomatology and/or global functioning, dietary intake, physical activity level, cardiorespiratory fitness, cognitive functioning, quality of life, eating behaviours, and nutrition knowledge of people living with a severe mental illness?

## Method

This systematic review and meta-analysis were pre-registered on the PROSPERO database (CRD42022323030) and conducted in accordance with the PRISMA statement (Supplement 1) to ensure comprehensive and transparent reporting.

### Search strategy

An electronic database search was completed from earliest record to August 2024 using Scopus, Medline, EMBASE, the Cochrane Central Register of Clinical Trials, PsycINFO and CINAHL, using key nutritional, cardiometabolic and psychiatric terminology (see Table S1 in supplementary information for full search strategy).

### Screening and selection process

Only peer-reviewed research articles available in English were included. Eligible studies included: specialist-delivered interventions by a dietitian and/or nutritionist or exercise professional (physiologist/physiotherapist) in people diagnosed with a severe mental illness (including schizophrenia, schizoaffective, schizophreniform, bipolar affective disorder, major depression with psychotic features and delusional disorder). The outcome inclusion criteria included metabolic and/or cardiometabolic health, mental illness symptomatology and/or global functioning, dietary intake, cardiorespiratory fitness, cognitive functioning, quality of life, eating behaviours/binge eating/intuitive eating and nutrition knowledge. Each search strategy was customised for the coding of each database used in order to encompass all fields and maximise sensitivity. Studies were required to have a control group to be included in the systematic review and meta-analysis (see Table S2 in supplementary information for full inclusion and exclusion criteria).

Four authors (TM, JK, RB, NB) independently reviewed the studies selected for inclusion, and those which did not satisfy the inclusion/exclusion criteria, were discussed and removed, with the reason for removal being recorded. Any discrepancies were referred to another author who was not involved in the initial review of studies.

### Data extraction

Study eligibility was assessed according to:

*1. Study selection*: author, year, control group, and specialist-delivered intervention.
*2. Study characteristics*: study design, country, sample size (*n*), type of intervention (group or individual, manual based or individualised, practical components or education), setting, frequency/intensity of sessions offered, and attrition rate.
*3. Participants*: age, sex, inpatient/outpatient, and mental illness.
*4. Study findings*: metabolic outcomes (weight, BMI, waist measurement, blood pressure, biochemistry). Secondary outcomes (mental illness symptomatology and/or global functioning, dietary intake, cardiorespiratory fitness, cognitive functioning, quality of life, eating behaviours/binge eating/intuitive eating, and nutrition knowledge).

### Assessment of quality of studies with risk-of-bias tool

Risk of bias was assessed using the Cochrane Risk of Bias tool for randomised controlled trials (RoB2) (Sterne et al., 2019), and the risk of bias in non-randomised studies of interventions tool (ROBINS-I) (Sterne et al., 2016) with particular attention to fidelity of intervention delivery. The overall risk of bias for randomised controlled trials was deemed high if risk was high in at least one domain; for non-randomised trials, the risk of bias was deemed serious when a serious risk was identified in at least one domain. Risk of bias was completed independently by two authors (TM & JK), any discrepancies were sent to a third author (RB) to rectify.

### Certainty of evidence

Certainty of evidence for each primary outcome was assessed by two reviewers (TM & JK) using the Grading of Recommendations Assessment, Development and Evaluation (GRADE) framework (Guyatt et al., 2008). Risk of bias, inconsistency, indirectness and imprecision are the four domains used in the GRADE assessment (further information can be found in Table s5 in the supplementary file).

### Statistical analysis

Where there was a sufficient number of studies that examined the same outcome measures, a meta-analysis using a DerSimonian–Laird random effects model was performed using Stata (StataCorp, 2023). To calculate the overall treatment effect, the end of the intervention data for both the intervention and control groups was used. To investigate the effect of any one individual study had on the overall model, a leave-one-out analysis was performed for analyses with sufficient studies. For analyses with ⩾10 included studies, Egger’s *t* test was conducted to assess potential publication bias. The *I*^2^ statistic was used to assess statistical heterogeneity between studies, with substantial heterogeneity considered where the *I*^2^ statistic was ⩾75%. All effect sizes were calculated using Hedge’s g, with an effect size of 0.2 considered small, 0.5 moderate and 0.8 considered large. Findings were considered statistically significant at *p* < 0.05.

## Results

### Study selection

**Figure 1.**
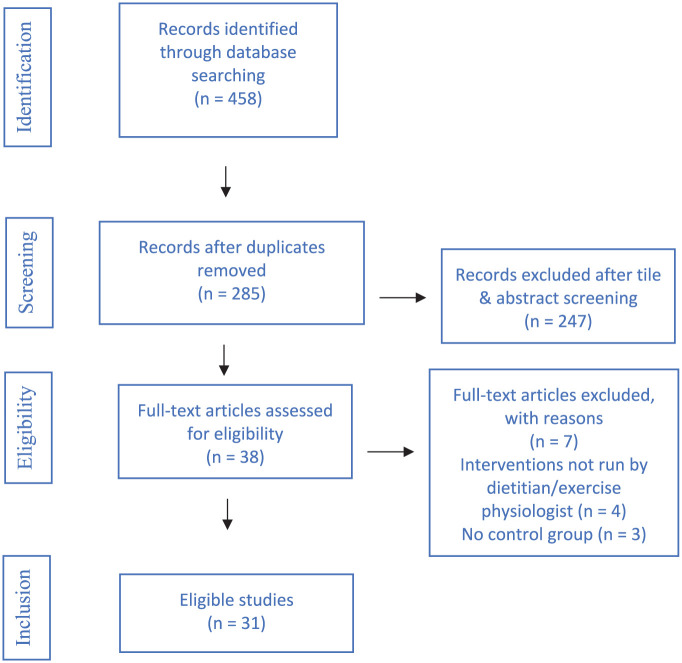
PRISMA search diagram (Page et al., 2021).

A database search was performed in August 2024. A total of 446 publications were retrieved from all sources and 161 duplicates were removed. Abstracts and titles of the remaining 285 publications were screened. Full-text versions were retrieved and reviewed by four authors for 38 articles, of which 31 were included and 7 were excluded due to interventions not being delivered by a dietitian/exercise professional (*n* = 4) or no control group (*n* = 3). Overall, findings in 31 studies were included in the systematic review and 24 studies were included in the meta-analysis. Figure 1 presents the PRISMA flow diagram (Page et al., 2021).

### Study characteristics

The study characteristics are shown in Table 1. All 31 studies were conducted from 2005 to 2024 and included 2738 participants (1522 intervention participants and 1216 controls). Seven studies were conducted in the United States of America (Brown et al., 2011; Erickson et al., 2016, 2017; Errichetti et al., 2020; Green et al., 2015; Jean-Baptiste et al., 2007; Massa et al., 2020), three studies were in Italy (Goracci et al., 2016; Magni et al., 2017; Scocco et al., 2006), and Norway (Brobakken et al., 2019, 2020; Heggelund et al., 2011), two studies were done in Canada (Marzolini et al., 2009; Romain et al., 2019), Australia (Curtis et al., 2016; Evans et al., 2005), Netherlands (Deenik et al., 2019; Scheewe et al., 2013) and Spain (Foguet-Boreu et al., 2022; Tous-Espelosin et al., 2024) and individual studies in Japan (Sugawara et al., 2018), Brazil (Attux et al., 2013), Germany (Cordes et al., 2014), Taiwan (Wu et al., 2007), South Korea (Kwon et al., 2006), Serbia (Curcic et al., 2017), Israel (Melamed et al., 2008), Denmark (Midtgaard et al., 2021), China (Zhang et al., 2023) and Switzerland (Gillhoff et al., 2010).

**Table 1.** Characteristics of the published trials included in our systematic review and meta-analysis.

	Participants **(Women); Age (Years), Mean (SD)**			Intervention **group**			Control **group**	
Author/s, Country	Control	Intervention	Diagnosis	Type, length, delivery mode, personnel	Description	Adherence/attendance	Description	Primary outcomes
Attux et al, Brazil	79 (33) 36.2 (9.9)	81 (31) 38.3 (10.7)	Schizophrenia	Lifestyle 12 weeks Group Dietitian Exercise specialist	12 sessions: introduction, four sessions on diet/nutrition; three sessions on physical activity; sessions on self- esteem, motivation, management of anxiety; session with relatives; wrap- up	Mean 9.1 (SD 3.5) sessions. 49 participants (72%) attended 8 or more sessions.	Standard care	Mean weight change: 3 months: intervention, –0.48 kg (95% CI, –0.65 to +1.13); control, +0.48 kg (95% CI, 0.13–0.83; *p* = 0.06). 6 months: intervention, –1.15 kg (95% CI, –2.11 to +0.19); control, +0.5 kg (95% CI, –0.42 to +1.42; *p* = 0.017)
Brobakken et al, Norway (A)	23 (12) 36 (12)	25 (8) 34 (10)	Schizophrenia	Exercise 12 Weeks Group Exercise specialist	Two sessions of Aerobic Interval Training (AIT) per week for 12 weeks.	At 12 weeks 16 (64%) and18 (78%) completed the intervention and control programme.	Standard care + 2 AIT sessions	Significant increase in VO2peak: Intervention – 2.77 L min – 2.94 L min (*p* ⩽ 0.01)
Brobakken et al, Norway (B)	23 (12) 36 (5)	25 (8) 34 (4)	Schizophrenia	Exercise 52 Weeks Group Exercise specialist	Two sessions of AIT per week for 52 weeks.	At 52 weeks 0 participants from control group and 15 (60%) participants from the intervention group successfully completed the programme.	Standard care + 2 AIT sessions	Significant increase in VO2peak: Intervention – 2.77 L min – 2.95 L min (*p* ⩽ 0.001)
Brown et al, United States of America	68 44.6 (10.9)	68 44.6 (10.9)	Severe mental illness	Lifestyle 52 weeks Mixed Dietitian Exercise specialist	12 month programme with 3 stages: 3-month intense phase (weekly 3 hr session on nutrition, physical activity, goal); 3-month maintenance phases (monthly meetings, weekly phone calls); 6 month intermittent support phase.	At 6 months – 89 (65%) were still enrolled. 21/68 (31%) enrolled in the intervention had discontinued. No information provided for 12 months.	Standard care	Mean weight change (completers only): Intervention – 2 kg; control – 0.4 kg (*p* = 0.005)
Cordes et al, Germany	38 (11) 35.8 (10.9)	36 (21) 38.2 (11.2)	Schizophrenia or schizoaffective disorder	Lifestyle 24 weeks Group Dietitian Exercise specialist	12 fortnightly sessions. Four modules. Module 1 (one session): assessment of eating and physical activity behaviours; module 2 (six sessions): nutrition–knowledge and skills; module 3 (one session); exercise–education; module 4 (four sessions)–behavioural techniques, stress management, coping strategies.	By 48 weeks 25 (34%) were still enrolled. 25/36 (69%) enrolled in the intervention had discontinued.	Standard care	Weight change: No significant differences between groups. Waist circumference (48 weeks): intervention, +4.6 cm (SD, 8.3); control, +10.1 cm (SD, 7.3) (*p* = 0.019). Fasting glucose (48 weeks): smaller increase in intervention group (*p* = 0.031)
Curcic et al, Croatia	40 (21) 41.75 (9.45)	40 (17) 39.95 (9.51)	Schizophrenia	Exercise 12 weeks Exercise Specialist	Four 45 minute sessions per week.	Not reported.	Standard care	VO2 max: Significant increase (*p* = 0.002) and compared with control group (*p* = 0.000)
Curtis et al, Australia	12 (2) 21.7 (1.9)	16 (9) 20 (2.3)	First episode psychosis	Lifestyle 12 weeks Mixed Dietitian Exercise specialist	Individualised programme with three components: health coaching – to help with adherence; dietetic support (education [weight management, labels, food quality], and practical skills [shopping, cooking]); and supervised physical activity sessions.	Mean, 8 diet sessions (range, 5–10); 11 physical activity sessions (range, 3 – 25)	Standard care	Mean weight change (12 weeks): Intervention, +1.8 kg (95% CI, −0.4 to +2.8); vs control, 7.8 kg (95% CI, 4.8–10.7; *p* < 0.001)
Deenik et al, Netherlands	43 (20) 58.6 (12.1)	65 (22) 52.2 (8.9)	Severe mental illnes + hospitalisation for at least 1 year.	Lifestyle 72 Weeks Group Dietitian	Improving daily structure: set time to wake by; three group meals per day; and an active day programme consisting of sports-related activities (e.g., walking, running, yoga), work-related activities (e.g., gardening and working in services within the hospital, psycho-education (e.g. about side effects, dietary habits) and daily living skills training (e.g. making a grocery list, cooking)	15 (12%) discontinued from the study	Standard care	Improved physical activity (*p* =< 0.05). Weight −4.18 kg (*p* = 0.04), abdominal girth 3.48 cm (*p* = 0.04), Sys BP –9.05 mmHg (*p* = 0.02), HDL cholesterol + 0.10mmol/l (*p* = 0.03)
Evans et al, Australia	22 (11) 33.6 (11.6)	29 (18) 34.6 (9.6)	Mental health patients prescribe Olanzapine	Nutrition 24 weeks Individual Dietitian	Six 1 hour nutrition education sessions covering healthy eating, exercise, label reading, energy density, high fibre diets, non-hungry eating and maintenance of healthy eating and changes in activity levels	At 12 weeks 6/29 (21%) had discontinued from the study. At 24 weeks 12/23 (52%) had discontinued from the study. Overall, at 24 weeks 32/51 (63%) had discontinued from the study.	Standard care + passive nutrition education	At 12 weeks, control group had gained significantly more weight (6.0 kg vs 2.0 kg, *p* = 0.002); BMI (2kgm/^2^ vs 0.7kgm/^2^, *p* = 0.03) At 24 weeks, control group had continued to gained significantly more weight (9.9 kg vs 2.0 kg, *p* = 0.013); BMI (3.2 kg/m^2^ vs 0.8 kg/m^2^, *p* = 0.017)
Erickson et al, United States of America (A)	48 (6) 49.6 (9.1)	60 (6) 49.7 (6.9)	Severe mental illness + antipsychotics + 7% weight gain or BMI > 25 kg/m^2^	Nutrition 52 weeks Mixed Dietitian	Eight weekly education classes (with monthly boosters): dietary monitoring; recommendations for calorie deficit (food, physical activity logs); individual coaching on nutrition, lifestyle; optional group physical activity classes	Completed programme: 25/60 (42%). Mean: 13.7 of 19 sessions	Standard care	Predicted mean weight change: Intervention, –4.6 kg; control, +0.6 kg
Erickson et al, United States of America (B)	42 (10) 50.4 (9)	62 (10) 51.9 (9.3)	Mental illness + antipsychotics + BMI > 25 kg/m^2^	Nutrition 52 weeks Mixed Dietitian	Eight weekly classes for the first 2 months, the remaining 10 months were monthly meeting. Classes covered: effects of medications; stress management; motivation, goal setting; diet, nutrition (mindful eating, portion sizes, calories, food groups, variety); physical activity. Food, physical activity journals. Participants also received individual nutrition counselling.	Completed initial programme: 8 weeks, 53/62 (86%), control group 50/59 (85%); 12 months, 33/62 (53%), control 17/59 (29%).	Standard care + sessions with study team including anthropometric measurements and health related education	Waist circumference, mean change: intervention, –1.04 cm; control, – 0.25 cm (*p* < 0.001) Body fat, mean change: Intervention, –0.4 percentage points; control, +0.2 percentage points (*p* = 0.038)
Errichetti et al, United States of America	167 (93) 40.7 (13.4)	249 (137) 41.0 (12.5)	Severe mental illness + hypertension OR obesity OR diabetes OR hypercholesterolemia	Nutrition 52 weeks Individual Dietitian	Referral to a dietitian–from care coordinator based on individual needs and co-morbid conditions	Retention rates: intervention 154/249 (62%); control 115/167 (69%)	Standard care	Intervention participants improved: Systolic blood pressure –3.86 mmHg (SD, 1.89), *p* = 0.04;, HbA1c: – 0.36% (SD, 0.11), *p* = 0.001
Foguet- Boreu et al, Spain	37 (17) 47.7 (10.3)	37 (17) 49.8 (11.4)	Severe mental disorders–schizophrenia, bipolar, depressive disorders, personality disorders and obsessive compulsory disorders	Nutrition 16 weeks Group Dietitian/Nutritionist	Weekly group sessions (5–10 people). Session lasted 90 minutes and covered food education to promote the consumption of fruit and vegetables.	Not reported	Standard care	Intervention group significant weight gain (87.0–89.0, *p* = 0.006), no differences observed in control group (81.3–81.4, *p* = 0.432). Significant increase in BMI (30.5–31.3, *p* = 0.000) and abdominal perimeter (104.9–105.1, *p* = 0.000) were observed. In control group the incident of MetS had increased (8–12, *p* = 0.005).
Goracci et al, Italy	79 (62) 48.8 (11.53)	81 (66) 49.45 (12.65)	Recurrent unipolar depression or bipolar disorder.	Lifestyle 12 weeks Individual Dietitian	1 individual session per week (45–60 minutes), coving a variety of topics including physical exercise and nutrition education.	Of the 64 participants who received the intervention, 48 completed the intervention (75%).	Standard care	Significant reduction in PHQ-9 scores, BMI and weight. BMI (*p* = 0.001) and weight (*p* = 0.001).
Gillhoff et al, Switzerland	24 (10) 48.9 (12.0)	26 (13) 48.1 (11.5)	Bipolar disorder	Lifestyle 20 weeks Group Nutrition and dietitian specialist	11 group sessions and weekly exercise classes.	No information provided.	Standard care (Waiter list)	Intention-to-treat analyses showed the intervention significantly reduced BMI (*p* = 0.03)
Green et al, United States of America	96 (69) 48.3 (9.7)	104 (75) 46.2 (11.4)	Antipsychotics + BMI > 27 kg/m^2^	Lifestyle 52 weeks Mixed Nutrition interventionist Exercise specialist	First 6 months: Weekly 2 h group meetings (incl. 20 min physical activity and dietary intake (participants taught to keep records of physical activity, intake including fruit, vegetable, low-fat dairy and fibre intake, and sleep. 6 months: Maintenance phase: monthly group meetings focusing on maintaining weight loss through problem solving and motivational enhancements. Participants also receive monthly individual phone calls.	91% of participants (181) completed 6 months, and 85% (170) completed 12 months. Average sessions attended by intervention participants were 14.5 (SD = 7.2) out of 24, maintenance sessions 2.7 (SD = 2.17) out of 6.	Standard care	Mean weight change:6 months: intervention lost 4.4 kg (95% CI, –6.96 kg to –1.78 kg) more than the control. 12 months: Intervention lost 2.6 kg (95% CI, –5.14 kg to –0.07 kg) more than control compared to baseline (*p* = 0.004). During maintenance (6–12 months): no difference.
Heggelund et al, Norway	12 (3) 30.5 (8.7)	7 (3) 38.9 (11.4)	Schizophrenia	Exercise 8 weeks Group Exercise specialist	High aerobic intensity training (HIT). HIT consisted of 4X4 min intervals with 3 min break periods, at 85%–95% and 70% of peak heart rate, respectively	Intervention group: Training sessions completed 85% (SD = 9%). Control group sessions completed 83% (SD = 6%) There were 6 participants who withdraw from the study however not included in results.	Standard care + computer games	VO_2peak_ – Intervention: 12% increase, 3.56 (SD = 0.68) l/min, (*p* ⩽ 0.001) compared to control. Net mechanical efficiency of walking – Intervention: 125 increase (22.2 (SD = 45) % (*p* + 0.005)
Jean-Baptiste et al, United States of America	9 (3) 40.7	9 (7) 52.4	Schizophrenia or schizoaffective disorder	Nutrition 32 weeks Group Dietitian	Weekly group sessions for 16 weeks – including nutrition education, physical activity enhancement, food provision (given $25 refund per week if purchased programme specific food, grocery store visits and cooking sessions and individual nutrition support. Monthly weight and blood pressure measured for 6 months post intervention.	4/18 (22%) did not complete the intervention phase.	Standard care (Waiter list)	At 16 weeks intervention group had a 2.9 kg weight loss v control 2.8 kg weight gain (*p* = 0.026). No differences were shown at the end when the control group had completed the intervention.
Kwon et al, South Korea	15 (10) 29.8 (6.1)	33 (23) 32 (9.2)	Schizophrenia or schizoaffective disorder + Olanzapine	Lifestyle 12 weeks Individual Dietitian Exercise specialist	Individual nutrition intervention including weight and body mass index, diet management (food diary, education about eating behaviours); Exercise management (exercise diary, education about daily lifestyle modification)); Safety; Quality of Life; and Compliance.	22/33 (66%) completed the intervention. All participants who completed the programme had a 80% compliance with diet management, however only 12 participants (36.4%) completed 80% of the exercise component.	Standard care	Significant differences in between intervention v control weight (–3.94 ± 3.63 kg vs. –1.48 ± 1.88 kg, *p* = 0.006) and BMI (–1.50 ± 1.34 vs. –0.59 ± 0.73, *p* = 0.007)
Magni et al, Italy	26 (11) 41.8 (10.1)	59 (32) 43.1 (9)	Mental health + antipsychotics	Lifestyle 16 weeks Group Nutrition & Exercise specialists	32 twice weekly 1 h sessions covering nutrition psychoeducation, Mediterranean diet-based diet plan and physical activity plan.	No information provided.	Standard care + food and nutrition information	BMI: Intervention, –1.9% (from 32.6 to 32.0); control, + 0.6% (from 35.0 to 35.2; *p* = 0.021).
Marzolini et al, Canada	6 (2) 43 (3)	7 (3) 46.7 (5)	Schizophrenia or schizoaffective disorder	Exercise 12 weeks Group Exercise specialists	Twice weekly exercise sessions for 12 weeks, plus encouraged to complete an additional session at home.	Mean attendance to exercise classes 72% (SD = 4.4%), range 54–87.5%. Completion of home sessions was 35%.	Standard care	6MWD: Intervention - + 27.7 (SD = 22.3 m; Control: 28.3 (SD = 26.6)m: Strength: 28.3 (SD = 8.8)%, (*p* = 0.01), control 12.5 (SD = 8.5)% (*p* = 0.2). Mental Health Inventory score: Intervention *p* ⩽ 0.03) with no change for control. Reduction in depressive symptoms correlated with greater adherence to exercise (*r* = −0.93, *p* = 0.02).
Massa et al, United States of America	17 53.18	21 52.52	Schizophrenia (male participants only)	Exercise 12 weeks Group Exercise specialist	3 sessions pre week (20–45 minutes a session). Sessions start at 20 minutes, increase by 5 minutes per week to max of 45 minutes.	9 (43%) completed the intervention, 6 (35%) completed the control group.	Standard care + stretching and balance training.	At 12 weeks both groups were slower at the 400 m walk; however, the intervention group had significantly less slowing (*p* = 0.011)
Melamed et al, Israel	31	28	Schizophrenia & BMI > 25 kg/m^2^	Lifestyle 12 Weeks Group Nutrition & Exercise specialists	Weekly nutrition counselling and group-based behaviour theraphy & 30 minute walks 5 times/week.	No information provided.	Standard care (wait list)	Significant improvement in QOL (*p* = 0.04). Significant reductions in BMI, (Intervention 34.1–31.3, control group 30.6–30.4) (*p* = 0.006)
Midtgaard et al, Denmark	12 (10) 27.5 (4.5)	13 (9) 23.8 (4.5)	First episode psychosis	Exercise 8 weeks (plus additional 8 weeks when control group commenced) Group Exercise specialist	1 hour sessions, 3 times per week.	76% of participants completed the first 8 weeks, this reduced to 52% at 16 weeks.	Standard care (wait list)	No significant changes were observed.
Romain et al, Cananda	28 (12) 32.12 (7.10)	38 (13) 29.70 (7.24)	Psychotic disorders + overweight	Exercise 24 weeks Individual Exercise specialist	Two sessions weekly	50% of participants completed the intervention.	Standard care (wait list)	Significant improvements in waist circumference (*p* = 0.04), PANNS negative (*p* = 0.01) and global functioning (*p* = 0.02) in the intervention groups.
Scheewee et al, Netherlands	32 (9) 30.1 (7.7)	31 (8) 29.2 (7,2)	Schizophrenia	Exercise 24 weeks Group Exercise specialists	Two X 1 hour sessions of exercise weekly	2/31 (7%) participants did not complete the intervention. 20 (65%) participants completed the required number of sessions (41 SD = 8). 2/32 (22%) did not complete the control. 19 (59%) participants completed the required number of sessions (42 SD = 7).	Standard care + creative and recreational activities	Per protocol analyses showed that exercise therapy reduced symptoms of schizophrenia (*p* = 0.001), depression (*p* = 0.012), need of care (*p* = 0.050), and increased cardiovascular fitness (*p* ⩽ 0.001) compared with occupational therapy.
Scocco et al, Italy	10 (2) 39.2 (9.9)	10 (7) 51.7 (12.4)	Schizophrenic disorders	Nutrition 24 weeks Mixed Nutritionist/Dietitian	Psychoeducational intervention + referral to a nutritionist.	18/20 (90%) participants completed the study	Standard care + psychoeducational intervention	Weight gain: Intervention (+0.99 + 3.34 kg) vs. Control (+2.96 + 3.08 kg); *p* ⩽ 0.03).
Sugawara et al, Japan	61 (26) 44.0 (10.3)	Group 1: 67 (36) 47.6 (9.6) Group 2: 61 (29) 46.6 (10.9)	Schizophrenia or schizoaffective disorder + Obesity	Nutrition 52 weeks Individual Dietitian	Two intervention groups: Group 1. Weight loss advice from psychiatrist. Group 2. Twelve monthly sessions with dietitian, covering – a balanced diet; food choices; food requirements; revision and discussion of food records	Group 1: 67/93 (72%); group 2, 61/87 (70%) completed the study	Standard care	Mean weight change: Greater decline for group 2 (–3.2 kg; SD, 4.5, *p* = <0.001) than group 1 (–0.4 kg; SD, 3.9, *p* = 0.434) and control (+0.5 kg; SD, 5.1), *p* = 0.458)
Tous-Espelosin et al, Spain	53 (12) 42.5 (9.9)	59 (13) 40.1 (10.9)	Schizophrenia	Exercise 20 weeks Not specified Exercise Specialist	3 sessions of HIIT per week for 20 weeks	Intervention group had to complete at a minimum 85 of sessions.	Standard care	Significant improvements in CRF (*p* ⩽ 0.05) and VO2peak (*p* ≤ 0.001.)
Wu et al, Taiwan	25 (14) 39 (6.7)	28 (17) 42.2 (7.5)	Schizophrenia + Obesity + clozapine	Lifestyle 24 weeks Mixed Dietitian	Diet that reduced calorie intake by 200 to 300 kcal per day (to 1,300 to 1,500 kcal per day for women and to 1,600 to 1,800 kcal per day for men) and a six-month regimen of regular physical activity in which participants used approximately 600 to 750 kcal per week (level walking and walking on stairs for 60 minutes three days per week).	100% completion rate for diet component, 90% of physical activity programme was completed.	Standard care	Intervention: significant decrease in body weight (−4.2 kg; SD 4.4; *p* ≤ 0.001), body mass index (−1.50; SD 1.66; *p* ≤ 0.001), waist circumference (−3.3 cm; SD 4.18; *p* ≤ 0.001), and hip circumference (−3.3 cm; SD 4.5; *p* ≤ 0.05) after three months and after six months. Triglyceride (*p* ≤ −0.05) and insulin-like growth factor-binding protein-3 (IGFBP-3) (*p* ≤ 0.05) decreased significantly only after six months.
Zhang et al, China	48 34.24 (10.96) *female only	47 37.02 (10.56) *female only	Obese women with schizophrenia	Nutrition 4 weeks Individual Dietitian	Calorie restricted diet, individualised to each participant. Weekly appointments.	No information provided.	Standard care + regular diet	Significant decrease in weight (2.38 ± 1.30 kg; *p* ≤ 0.001), BMI (0.94 ± 0.52 kg/m2; *p* ≤ 0.001), waist circumference (4.34 ± 2.75 cm; *p* ≤ 0.001)

Twenty-four studies were randomised controlled trials (Attux et al., 2013; Brobakken et al., 2019, 2020; Brown et al., 2011; Cordes et al., 2014; Curcic et al., 2017; Erickson et al., 2016, 2017; Errichetti et al., 2020; Evans et al., 2005; Foguet-Boreu et al., 2022; Gillhoff et al., 2010; Goracci et al., 2016; Green et al., 2015; Kwon et al., 2006; Massa et al., 2020; Midtgaard et al., 2021; Romain et al., 2019; Scheewe et al., 2013; Scocco et al., 2006; Sugawara et al., 2018; Tous-Espelosin et al., 2024; Wu et al., 2007; Zhang et al., 2023), five were non-randomised controlled trials (Curtis et al., 2016; Deenik et al., 2019; Heggelund et al., 2011; Magni et al., 2017; Melamed et al., 2008) and two were pilot studies (Jean-Baptiste et al., 2007; Marzolini et al., 2009). Thirty studies included an intervention and control arm, of these 30 studies, 5 were cross-over designs (Gillhoff et al., 2010; Jean-Baptiste et al., 2007; Melamed et al., 2008; Midtgaard et al., 2021; Romain et al., 2019) and 25 were parallel designs (Attux et al., 2013; Brobakken et al., 2020; Brobakken et al., 2019; Brown et al., 2011; Cordes et al., 2014; Curcic et al., 2017; Curtis et al., 2016; Deenik et al., 2019; Erickson et al., 2016, 2017; Errichetti et al., 2020; Evans et al., 2005; Foguet-Boreu et al., 2022; Goracci et al., 2016; Green et al., 2015; Heggelund et al., 2011; Kwon et al., 2006; Magni et al., 2017; Marzolini et al., 2009; Massa et al., 2020; Scheewe et al., 2013; Scocco et al., 2006; Tous-Espelosin et al., 2024; Wu et al., 2007; Zhang et al., 2023). One study was a three-arm trial (two intervention arms and one control group) (Sugawara et al., 2018). Twelve studies had a lifestyle intervention which included both nutrition and exercise (Attux et al., 2013; Brown et al., 2011; Cordes et al., 2014; Curtis et al., 2016; Deenik et al., 2019; Gillhoff et al., 2010; Goracci et al., 2016; Green et al., 2015; Kwon et al., 2006; Magni et al., 2017; Melamed et al., 2008; Wu et al., 2007), nine studies included a nutrition intervention without exercise (Erickson et al., 2017; Erickson et al., 2016; Errichetti et al., 2020; Evans et al., 2005; Foguet-Boreu et al., 2022; Jean-Baptiste et al., 2007; Scocco et al., 2006; Sugawara et al., 2018; Zhang et al., 2023), and 10 studies included an exercise intervention without nutrition (Brobakken et al., 2020; Brobakken et al., 2019; Curcic et al., 2017; Heggelund et al., 2011; Marzolini et al., 2009; Massa et al., 2020; Midtgaard et al., 2021; Romain et al., 2019; Scheewe et al., 2013; Tous-Espelosin et al., 2024).

Twelve studies enrolled participants diagnosed with schizophrenia and/or schizoaffective disorders (Attux et al., 2013; Brobakken et al., 2019, 2020; Cordes et al., 2014; Curcic et al., 2017; Heggelund et al., 2011; Jean-Baptiste et al., 2007; Marzolini et al., 2009; Massa et al., 2020; Scheewe et al., 2013; Scocco et al., 2006; Tous-Espelosin et al., 2024). Four included participants diagnosed with a severe mental illness (Brown et al., 2011; Deenik et al., 2019; Foguet-Boreu et al., 2022; Romain et al., 2019). Similarly, two studies included participants with schizophrenia and/or schizoaffective disorder and who were prescribed olanzapine (Kwon et al., 2006; Wu et al., 2007). Persons with bipolar and recurrent depression were invited to participate in two studies (Gillhoff et al., 2010; Goracci et al., 2016). Participants with first episode psychosis were invited to participate in two papers (Curtis et al., 2016; Midtgaard et al., 2021). Two studies included participants who had been prescribed antipsychotics (Evans et al., 2005; Magni et al., 2017). Two other studies included participants diagnosed with a severe mental illness, who had been prescribed antipsychotics and had either BMI ⩾ 5 kg/m^2^ or 7% weight gain since the commencement of antipsychotic drugs (Erickson et al., 2017; Erickson et al., 2016). Participants with a BMI ⩾ 25 kg/m^2^ and diagnosed with schizophrenia were included in one study (Melamed et al., 2008). Participants who were obese and diagnosed with schizophrenia and/or schizoaffective disorder were included in one study (Sugawara et al., 2018). One studied included persons who had a BMI ⩾27 kg/m^2^ and were prescribed antipsychotics (Green et al., 2015). Women with obesity who had been diagnosed with schizophrenia were studied in one study (Zhang et al., 2023). Participants with a severe mental health diagnosis and one or more of the following obesity, hypertension, diabetes or hypercholesterolemia were included in one study (Errichetti et al., 2020). Study sample sizes (including intervention and control arms) ranged from 13 participants (Marzolini et al., 2009) to 416 participants (Errichetti et al., 2020).

Study interventions varied; one study had a 4-week intervention (Zhang et al., 2023), another had an intervention of 8 weeks (Heggelund et al., 2011), nine studies had 12-week interventions (Attux et al., 2013; Brobakken et al., 2019; Curcic et al., 2017; Curtis et al., 2016; Goracci et al., 2016; Kwon et al., 2006; Marzolini et al., 2009; Massa et al., 2020; Melamed et al., 2008), three had 16-week interventions (Foguet-Boreu et al., 2022; Magni et al., 2017; Midtgaard et al., 2021), two had 20-week interventions (Gillhoff et al., 2010; Tous-Espelosin et al., 2024), six had 24-week interventions (Cordes et al., 2014; Evans et al., 2005; Romain et al., 2019; Scheewe et al., 2013; Scocco et al., 2006; Wu et al., 2007), one had 32 week intervention (Jean-Baptiste et al., 2007) and six had 52-week interventions (Brown et al., 2011; Erickson et al., 2016, 2017; Errichetti et al., 2020; Green et al., 2015; Sugawara et al., 2018). Finally one study used data from the previous 72 weeks (Deenik et al., 2019). Two studies had a lead in time prior to commencing the interventions of 4 weeks (Cordes et al., 2014) and 12 weeks (Evans et al., 2005). Four studies had participant data collected 6 months after the intervention had been completed (Attux et al., 2013; Cordes et al., 2014; Evans et al., 2005; Jean-Baptiste et al., 2007), another study had a follow up period of 20 weeks (Massa et al., 2020).

Thirty studies included female and male participants, one study included females only (Zhang et al., 2023). Thirty studies included adult participants, seven studies specified the intervention range with three including 18–65 years (Brobakken et al., 2019; Cordes et al., 2014; Wu et al., 2007), one 18–55 years (Romain et al., 2019), two included 18–70 years (Erickson et al., 2017; Gillhoff et al., 2010) and one included 19–64 years (Kwon et al., 2006). The final study included participants aged from 14 to 25 years (Curtis et al., 2016). Six studies reported the ethnicity of participants (Curtis et al., 2016; Erickson et al., 2017; Erickson et al., 2016; Green et al., 2015; Romain et al., 2019; Scheewe et al., 2013; Twenty-three studies enrolled participants from the community and/or outpatient setting (Attux et al., 2013; Brobakken et al., 2019, 2020; Brown et al., 2011; Curtis et al., 2016; Erickson et al., 2016, 2017; Errichetti et al., 2020; Evans et al., 2005; Foguet-Boreu et al., 2022; Gillhoff et al., 2010; Goracci et al., 2016; Green et al., 2015; Jean-Baptiste et al., 2007; Kwon et al., 2006; Marzolini et al., 2009; Massa et al., 2020; Midtgaard et al., 2021; Romain et al., 2019; Scheewe et al., 2013; Scocco et al., 2006; Sugawara et al., 2018; Tous-Espelosin et al., 2024). Seven studies enrolled participants from an inpatient setting (Cordes et al., 2014; Curcic et al., 2017; Deenik et al., 2019; Heggelund et al., 2011; Melamed et al., 2008; Wu et al., 2007; Zhang et al., 2023) and one study enrolled participants from both inpatient and outpatient settings (Magni et al., 2017). Attrition rates varied from 0% to 61%.

A variety of outcome measures (Table 1) were reported throughout the 31 studies. Twenty-three studies included weight (Attux et al., 2013; Brobakken et al., 2019, 2020; Brown et al., 2011; Cordes et al., 2014; Curtis et al., 2016; Deenik et al., 2019; Erickson et al., 2016; Evans et al., 2005; Foguet-Boreu et al., 2022; Gillhoff et al., 2010; Goracci et al., 2016; Green et al., 2015; Jean-Baptiste et al., 2007; Kwon et al., 2006; Marzolini et al., 2009; Melamed et al., 2008; Romain et al., 2019; Scocco et al., 2006; Sugawara et al., 2018; Tous-Espelosin et al., 2024; Wu et al., 2007; Zhang et al., 2023). BMI was reported in 21 studies (Brobakken et al., 2020; Brobakken et al., 2019; Cordes et al., 2014; Curtis et al., 2016; Erickson et al., 2016, 2017; Errichetti et al., 2020; Evans et al., 2005; Foguet-Boreu et al., 2022; Gillhoff et al., 2010; Goracci et al., 2016; Green et al., 2015; Magni et al., 2017; Marzolini et al., 2009; Massa et al., 2020; Melamed et al., 2008; Romain et al., 2019; Sugawara et al., 2018; Tous-Espelosin et al., 2024; Wu et al., 2007; Zhang et al., 2023). Seventeen studies included blood pressure (Brobakken et al., 2019, 2020; Cordes et al., 2014; Curtis et al., 2016; Deenik et al., 2019; Erickson et al., 2016; Errichetti et al., 2020; Foguet-Boreu et al., 2022; Gillhoff et al., 2010; Goracci et al., 2016; Green et al., 2015; Marzolini et al., 2009; Midtgaard et al., 2021; Romain et al., 2019; Sugawara et al., 2018; Tous-Espelosin et al., 2024; Zhang et al., 2023). Seventeen studies measured waist and/or hip circumference (Attux et al., 2013; Brobakken et al., 2020; Cordes et al., 2014; Curtis et al., 2016; Erickson et al., 2016, 2017; Evans et al., 2005; Gillhoff et al., 2010; Goracci et al., 2016; Magni et al., 2017; Marzolini et al., 2009; Romain et al., 2019; Scheewe et al., 2013; Sugawara et al., 2018; Tous-Espelosin et al., 2024; Wu et al., 2007; Zhang et al., 2023). Thirteen studies included triglycerides (TG) (Brobakken et al., 2019, 2020; Cordes et al., 2014; Curtis et al., 2016; Erickson et al., 2016; Foguet-Boreu et al., 2022; Gillhoff et al., 2010; Green et al., 2015; Jean-Baptiste et al., 2007; Romain et al., 2019; Sugawara et al., 2018; Tous-Espelosin et al., 2024; Zhang et al., 2023), and high-density lipoprotein cholesterol (HDL-C) (Brobakken et al., 2019, 2020; Curtis et al., 2016; Deenik et al., 2019; Erickson et al., 2016; Foguet-Boreu et al., 2022; Gillhoff et al., 2010; Green et al., 2015; Kwon et al., 2006; Romain et al., 2019; Sugawara et al., 2018; Tous-Espelosin et al., 2024; Zhang et al., 2023) respectively. Ten studies included glucose (Brobakken et al., 2019, 2020; Cordes et al., 2014; Curtis et al., 2016; Erickson et al., 2016; Green et al., 2015; Jean-Baptiste et al., 2007; Romain et al., 2019; Sugawara et al., 2018; Tous-Espelosin et al., 2024). Cholesterol (Brobakken et al., 2019, 2020; Cordes et al., 2014; Erickson et al., 2016; Errichetti et al., 2020; Foguet-Boreu et al., 2022; Gillhoff et al., 2010; Romain et al., 2019; Tous-Espelosin et al., 2024; Wu et al., 2007; Zhang et al., 2023) and low-density lipoprotein cholesterol (LDL-C) (Brobakken et al., 2019, 2020; Curtis et al., 2016; Erickson et al., 2016; Foguet-Boreu et al., 2022; Gillhoff et al., 2010; Green et al., 2015; Kwon et al., 2006; Romain et al., 2019; Tous-Espelosin et al., 2024; Zhang et al., 2023) were reported in 11 studies.

Outcomes related to improving overall mental health symptomology and/or quality of life, physical activity levels and nutrition knowledge were observed in a smaller number of studies. Depressive symptoms were report in three studies (Heggelund et al., 2011; Marzolini et al., 2009; Scheewe et al., 2013). Positive and Negative Syndrome Symptoms (PANSS) was include in 10 studies (Attux et al., 2013; Cordes et al., 2014; Curcic et al., 2017; Heggelund et al., 2011; Kwon et al., 2006; Magni et al., 2017; Massa et al., 2020; Romain et al., 2019; Scheewe et al., 2013; Tous-Espelosin et al., 2024). Global functioning was reported in one study (Romain et al., 2019), VO2peak or VO2max was included by seven studies (Brobakken et al., 2020; Curcic et al., 2017; Heggelund et al., 2011; Massa et al., 2020; Midtgaard et al., 2021; Scheewe et al., 2013; Tous-Espelosin et al., 2024). Heart rate was included in five studies (Brobakken et al., 2020; Cordes et al., 2014; Goracci et al., 2016; Kwon et al., 2006; Midtgaard et al., 2021). A study by Kwon et al. (2006) also included Physical Health Score. Marzolini et al. (2009) included a 6-minute walk distance (6MWD), muscle strength, and the mental health inventory score (MHIS). Cardiorespiratory fitness was included in 2 studies (Scheewe et al., 2013; Tous-Espelosin et al., 2024). Metabolic syndrome (MetS) was reported in three studies (Foguet-Boreu et al., 2022; Scheewe et al., 2013; Sugawara et al., 2018), and quality of life (QoL) was also reported in three studies (Evans et al., 2005; Melamed et al., 2008; Romain et al., 2019).

Fifteen of the 23 studies (65%) that reported on body weight reported a significant between-group difference (Brobakken et al., 2019; Brown et al., 2011; Curtis et al., 2016; Deenik et al., 2019; Erickson et al., 2016; Evans et al., 2005; Goracci et al., 2016; Green et al., 2015; Jean-Baptiste et al., 2007; Kwon et al., 2006; Melamed et al., 2008; Sugawara et al., 2018; Tous-Espelosin et al., 2024; Wu et al., 2007; Zhang et al., 2023). Fifteen of the 21 studies (71%) that reported on BMI found a significant between-group difference (Brobakken et al., 2019; Curtis et al., 2016; Erickson et al., 2016, 2017; Evans et al., 2005; Gillhoff et al., 2010; Goracci et al., 2016; Green et al., 2015; Kwon et al., 2006; Magni et al., 2017; Melamed et al., 2008; Sugawara et al., 2018; Tous-Espelosin et al., 2024; Wu et al., 2007; Zhang et al., 2023). Six out of 17 studies (35%) had a significant effect on waist circumference (Cordes et al., 2014; Curtis et al., 2016; Erickson et al., 2016; Romain et al., 2019; Sugawara et al., 2018; Wu et al., 2007). Significant improvements in PANSS scores were shown in four studies (Cordes et al., 2014; Curcic et al., 2017; Romain et al., 2019; Scheewe et al., 2013). Four studies showed significant improvements in VO2 scores (Brobakken et al., 2019, 2020; Curcic et al., 2017; Tous-Espelosin et al., 2024).

Research by Deenik et al. (2019) also reported significant improvements in intervention participants’ HDL-C and systolic blood pressure. Significant improvements in participant cardiovascular fitness were reported by two studies (Brobakken et al., 2020; Scheewe et al., 2013). There were also significant improvements in MHIS (Marzolini et al., 2009), QoL (Melamed et al., 2008), Global Functioning (Romain et al., 2019), and MetS (Foguet-Boreu et al., 2022). Significant effects on weight were identified in one study at the 6-month follow-up, (Attux et al., 2013), with no significant changes observed at the 3-month mark. After 4 months, the intervention by Cordes et al (2014) reported a significant effect on glucose, with improved glucose control in the intervention participants when compared to the control participants. TG and insulin-like growth factor-binding protein-3 were significantly decreased in the study by Wu et al. (2007). These results were only seen at 6 months and not at 3 months.

## Risk of bias

Two studies were deemed to have some risk of bias (Green et al., 2014; Marzolini et al., 2009), 29 studies were deemed to be high risk (Attux et al., 2013; Brobakken et al., 2019, 2020; Brown et al., 2011; Cordes et al., 2014; Curcic et al., 2017; Curtis et al., 2016; Deenik et al., 2019; Erickson et al., 2016, 2017; Errichetti et al., 2020; Evans et al., 2005; Foguet-Boreu et al., 2022; Gillhoff et al., 2010; Goracci et al., 2016; Heggelund et al., 2011; Jean-Baptiste et al., 2007; Kwon et al., 2006; Magni et al., 2017; Massa et al., 2020; Melamed et al., 2008; Midtgaard et al., 2021; Romain et al., 2019; Scheewe et al., 2013; Scocco et al., 2006; Sugawara et al., 2018; Tous-Espelosin et al., 2024; Wu et al., 2007; Zhang et al., 2023). Risk of bias due to fidelity and double-blinding was low to high in all studies, only one study (Erickson et al., 2017) specifiically reporting how they minimised bias due to fidelity. Risks were generally associated with low adherence rates by participants to intervention measures (see Tables S3 and S4, and Figure S1 and S2, supplementary information for more detail). Certainty of evidence was found to be low for all primary outcomes (see Table S5, in the supplementary information for more detail).

### Meta-analysis

Twenty-four papers were included in the meta-analysis. Seven papers were excluded from the analysis due to incomplete data being published and authors unable to access data from the paper’s authors. Meta-analyses were completed on nutrition only interventions (*n* = 5), exercise only intervention (*n* = 9) and combined nutrition and exercise interventions (*n* = 10). Outcomes included in the meta-analyses were BMI, weight, waist, blood pressure (diastolic and systolic), cholesterol, TG, HDL-C, LDL-C. There were insufficient papers to run meta-analysis on secondary outcomes.

**Figure 2.**
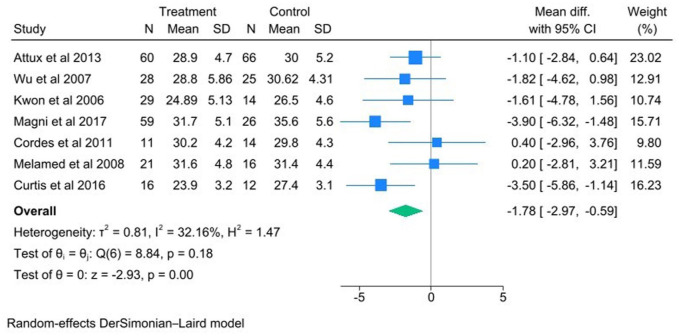
Forrest plot for trials that assessed the Impact of combined nutrition and exercise intervention on BMI.

**Figure 3.**
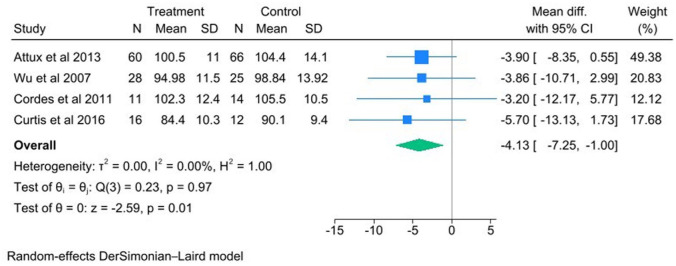
Forrest plot for trials that assessed the impact of combined nutrition and exercise intervention on waist.

Meta-analysis of combined nutrition and exercise interventions revealed a positive-effects on BMI (Figure 2; Mean diff = −1.78 [95% CI −2.97 to −0.59], *p* = 0.0); 7 studies were included in the meta-analysis with 208 intervention participants and 161 control participants. A positive effect was also revealed on waist circumference (Figure 3: Mean diff = −4.13 [95%CI −7.25 to −1.00], *p* = 0.01); 4 studies were included in the analysis with 115 intervention participants and 117 control participants. Waist circumference showed no heterogeneity between studies with *I*^2^ = 0% and BMI had a moderate heterogeneity (*I*^2^ = 32.16%). There was also a favourable impact on weight (Hedges g = −3.46 [95% CI −8.74 to 1.83], *p* = 0.20), cholesterol (Hedges g = −0.16 [95% CI −0.38 to 0.07], *p* = 0.17), glucose (Mean diff = −0.20[95% CI −0.41 to 0.02], *p* = 0.07), HDL-C (Hedges g = −0.18 [−0.43 to 0.06], *p* = 0.14)., TG (Hedges g = −0.28 [95% CI −0.55 to −0.00], *p* = 0.05), and blood pressure, both diastolic (Mean diff = −1.47 [−4.41 to 1.47], *p* = 0.33). and systolic (Mean diff = −7.65 [95% CI −20.08 to 4.80], *p* = 0.23)., respectively.

In nutrition-only intervention studies, the meta-analysis revealed a positive-effect on systolic blood pressure (Mean diff = –6.14 [95% CI −12.02 to –0.26], *p* = 0.04); 4 studies were included with 212 intervention participants and 216 control participants. There was a medium level of heterogeneity shown be between studies (*I*^2^ = 78.46%). The meta-analysis results also showed there was a favourable impact on weight (Mean diff = –2.37 [95% CI –4.96 to –0.21], *p* = 0.07), BMI (Mean Diff = –0.45 [95% CI –1.19 to –0.29], *p* = 0.10), waist (Mean diff = –2.29 [95% CI –4.99 to –0.42], *p* = 0.10) and diastolic blood pressure (Mean diff = –1.76 [95% CI –3.78 to –0.25], *p* = 0.09). There were no effects found for lipids or glucose. In exercise-only intervention studies, there were no significant effects identified, some analyses did show a favourable impact on weight (Mean diff = –2.07 [95% CI –7.46 to –3.33], *p* = 0.45)., waist (Mean diff = –3.14 [95% CI –9.11 to –2.82], *p* = 0.30), and TG (Hedges g = –0.13 [95% CI –0.71 to –0.44], *p* = 0.65), there were no effects found for blood pressure or glucose (see Figures S3–S5 for all meta-analyses, supplementary information for more detail). Due to insufficient numbers meta-analyses were not run on VO2max/VO2peak. Despite some asymmetry in the funnel plot, publication bias was not significant (see Figure S6, supplementary information for more detail). The leave-out method was also used in all meta-analyses to help identify influential studies, ensure reliability of results and identify any direct outliers.

## Discussion

This is the first systematic review and meta-analysis to examine the effects of specialist (dietitian and exercise professional or equivalent) delivered interventions with people diagnosed with a severe mental illness. The evidence from this paper demonstrates the importance of interventions which include a nutrition and/or exercise component and highlights the longer lasting results on physical health outcomes when combined with pharmacological and psychological interventions used in people managing a severe mental illness. The results indicated a wide range of outcomes from stand-alone interventions in either nutrition or exercise. From the meta-analysis nutrition interventions showed significant improvements on systolic blood pressure and there were no significant improvements shown in exercise interventions. However, individual studies did show significant results suggesting there is some evidence for stand-alone intervention. Nevertheless, combined lifestyle interventions (nutrition and exercise) demonstrated stronger results. The meta-analysis showed significant improvements in BMI, waist circumferences and a positive effect on weight. Similar to the stand-alone interventions some combined interventions have significant effects on weight. Overall, combined lifestyle interventions have shown they can enhance an individual’s physical health, which may be negatively impacted by antipsychotic medication used in the treatment of people living with a severe mental illness.

Significant impacts on metabolic outcomes including blood pressure, glucose, insulin and fatty acids (TG, HDL-C, LDL-C, Total Cholesterol) were identified in a smaller number of studies where duration of the intervention and adherence rates could have an impact. Fourteen of the studies included in the review had an intervention duration of 4 months or less, with only 6 studies having an intervention duration of 12 months. Adherence rates varied greatly throughout the studies, with some as low as 39%. Adherence rates may have been impacted by study duration and the number of overall participants.

Diet and exercise interventions are well established in the general population for reducing the burden of disease (Nitschke et al., 2022) and more recently have become a focus point for improving the physical health outcomes in the mental health population; however, the research is still limited in this population (Firth et al., 2019). The burden of chronic disease is an ongoing worldwide concern (Anderson and Durstine, 2019). Research has shown that people managing a severe mental illness have a lower life expectancy than that of the general population and are also experiencing chronic diseases at a higher proportion than that of the general population, this is secondary to the negative consequences of the antipsychotic medication (Firth et al., 2019; Morgan et al., 2012). People living with a mental illness are often overlooked or not heard when it comes to their health concerns, as a result are experiencing poorer health outcomes which are already at a higher risk secondary to the inequalities they already face (Liberati et al., 2025; Monk et al., 2024), Two of the contributing factors to the increased burden of disease faced by those living with a mental health condition are physical inactivity and access to nutritious food (Kettle et al., 2022; Segal and Opie, 2015). In the general population, given the complexities of diet and exercise, a dietitian or exercise specialist would form part of the multidisciplinary team (Allen et al., 2014; Kettle et al., 2022; Segal and Opie, 2015). In comparison, currently, people experiencing severe mental health challenges often do not have access to a dietitian or exercise specialist as part of the standard care plan even though these interventions have shown been effective in the general community (Firth et al., 2019; Nitschke et al., 2022). This paper highlights the importance of including dietitians and exercise specialists into standard mental health care among the multi-level interventions that will improve the physical health care and outcomes of people living with a mental health condition.

The evidence from this review found significant improvements with weight (66% of studies which included weight as an outcome); BMI (66% of studies which included BMI as an outcome); and waist circumference (35% of studies which included waist circumference as an outcome), and the results from some of studies also suggests there can be significant improvements in individual overall mental health; cardiorespiratory fitness and quality of life with the inclusion of nutrition and/or exercise component into care plans. The evidence from this research is supported by recent research that has found similar results in similar study populations, which have shown evidence to support the implementation of nutrition and/or exercise interventions as part of standard care of people diagnosed with a severe mental illness (Bradley et al., 2022; Rocks et al., 2022). Research by Rocks et al found some evidence for nutrition interventions for improving metabolic syndrome risk factors in people living with a severe mental illness. Their research suggested that intervention may be more effective when delivered by a specialist and on an individual basis (Rocks et al., 2022). Similarly, comparable lifestyle intervention for people living with a severe mental illness can have small but significant improvements on weight, BMI, waist circumference, physical activity and vegetable consumption (Bradley et al., 2022).

There was however no evidence to suggest improvements to dietary intake, eating behaviours and nutrition knowledge. Due to the lack of studies which included these secondary outcomes, these outcomes were unable to be systematically assessed. The overall certainty of evidence is limited by the limited reporting on intervention fidelity. Without consistent fidelity assessments, is difficult to determine whether the observed effects reflect the intended intervention or variations in implementation.

## Strengths and limitations

There were several strengths of this study. First, this was the first systematic review and meta-analysis to examine the effects of specialist (dietitian and exercise professional or equivalent) delivered interventions with people diagnosed with a severe mental illness. This research provides evidence to support the implementation of interventions including nutrition and/or exercise as part of standard care of people living with a severe mental illness and further supports claims from recent research in this area (Bradley et al., 2022; Rocks et al., 2022). This important finding should be considered in the development of health service workforce plans and relevant policies to ensure that people managing a severe mental illness are able to access a dietitian and exercise professional as part of their routine care in all health services. A limitation of this review was a small number of studies being included in the meta-analysis which suggests that the estimated effect sizes may not properly reflect the findings of all publications included in the review. A further limitation is the high risk of bias of all studies due to the lack of double-blinding and fidelity of study designs. Based on the results, further research should be conducted to determine whether nutrition-only, exercise-only or combined interventions have overall (non-metabolic) benefits, does the duration and frequency of the interventions, inpatient or outpatient settings, and individual versus group programmes have different effects. It is recommended that future research look at factors that influence adherence to lifestyle programmes including roles of peer support workers or peer support workers with lived experience, programmes that have been co-designed with participants. Further research should also consider including more younger people diagnosed with a severe mental illness and whether commencing interventions closer to the date of diagnosis is more beneficial for protecting the physical health of people living with a severe mental illness.

## Conclusion

The results of this review provide evidence to support the inclusion of combined nutrition and exercise programmes in the standard care of people living with a severe mental illness. The evidence from this review suggests weight; BMI; waist circumference; overall mental health; cardiorespiratory fitness and quality of life can be improved with the inclusion of lifestyle interventions, led by specialists, into standard mental health care plans. These finding support the inclusions of lifestyle intervention into mental health care to improve the physical health outcomes of those living with a mental illness; however, more research is needed to determine the duration of intervention and factors to increase adherence to programmes and when interventions should be commenced.

## Supplemental Material

sj-docx-1-anp-10.1177_00048674251384054 – Supplemental material for Effectiveness of specialist-delivered interventions in severe mental illness: A systematic review and meta-analysis Supplemental material, sj-docx-1-anp-10.1177_00048674251384054 for Effectiveness of specialist-delivered interventions in severe mental illness: A systematic review and meta-analysis by Tamieka Mawer, Scott Teasdale, Rachel Bacon, Nicholas Brown, Andrew McKune and Jane Kellett in Australian & New Zealand Journal of Psychiatry
