# Supplementary material for: Effectiveness of specialist-delivered interventions in severe mental illness: A systematic review and meta-analysis
Source: Aust N Z J Psychiatry. 2025 Oct 22;60(4):348–66. doi: 10.1177/00048674251384054 (PMC12999995; doi:10.1177/00048674251384054)

**Table S1. Search strategy.**

| Data bases searched: Scopus, Medline, EMBASE, the Cochrane Central Register of Clinical Trials, PsycINFO and CINAHL  Last search was August 2024 | Severe mental illness OR serious mental illness OR schizophrenia OR schizophrenic or schizoaffective disorder OR psychosis OR psychotic OR psychotic disorder OR manic depression OR bipolar OR delusional disorder OR early onset psychosis. |
| --- | --- |
|  | **Nutrition OR diet OR food OR eating OR weight management** |
|  | **Physical activity OR exercise OR lifestyle (life style) OR fitness** |
|  | **Nutritionist OR dietitian (dietician) OR nutrition specialist OR registered nutritionist OR registered dietitian (dietician)** |
|  | **Exercise physiologist OR exercise scientist OR physiotherapist or exercise specialist** |

**Table S2. Inclusion and exclusion criteria using the Participant, Intervention, Comparator, Outcome, Source framework.**

| Component | Inclusion criteria | Exclusion criteria |
| --- | --- | --- |
| Population | Studies in which the participants have been diagnosed with a severe mental illness. | Studies in which the participants have not been diagnosed with a severe mental illness.  Studies in which participants have been diagnosed with feeding or eating disorder. |
| Intervention | Randomised controlled trials or trials with controlled groups with interventions delivered by specialist (Dietitian or exercise professionals or equivalent. Interventions will either be combined nutrition and exercise, stand-alone nutrition or stand-alone exercise. | All other types of studies |
| Comparator | Studies with ‘treatment as usual’ or ‘standard care’ (e.g., medication and/or other therapy). | Studies not using ‘treatment as usual’ or ‘standard care’ (e.g., medication and/or other therapy) as the comparator. |
| Outcome | Studies with the following primary outcomes: body weight or composition, BMI, blood lipids (cholesterol, HDL, LDL), blood pressure, blood glucose and triglycerides. | Studies with any other primary outcomes. |

**Risk of Bias**

**Table S3: Traffic light plot of the domain-level assessment for the twenty-six randomised controlled trials.**

| Paper | D1 | D2 | D3 | D4 | D5 | Overall |
| --- | --- | --- | --- | --- | --- | --- |
| Attux 2013 |  |  |  |  |  |  |
| Brown 2011 |  |  |  |  |  |  |
| Brobakken 2019 |  |  |  |  |  |  |
| Brobakken 2020 |  |  |  |  |  |  |
| Cordes 2014 |  |  |  |  |  |  |
| Curic 2017 |  |  |  |  |  |  |
| Erickson 2016 |  |  |  |  |  |  |
| Erickson 2017 |  |  |  |  |  |  |
| Errichetti 2020 |  |  |  |  |  |  |
| Evans 2005 |  |  |  |  |  |  |
| Foguet-Boreu 2022 |  |  |  |  |  |  |
| Gillhoff 2010 |  |  |  |  |  |  |
| Goracci 2016 |  |  |  |  |  |  |
| Green 2015 |  |  |  |  |  |  |
| Jean-Baptist 2007 |  |  |  |  |  |  |
| Kwon 2006 |  |  |  |  |  |  |
| Marzolini 2009 |  |  |  |  |  |  |
| Massa 2020 |  |  |  |  |  |  |
| Midtgaard 2021 |  |  |  |  |  |  |
| Romain 2018 |  |  |  |  |  |  |
| Scheewe 2013 |  |  |  |  |  |  |
| Scocco 2006 |  |  |  |  |  |  |
| Sugawara 2018 |  |  |  |  |  |  |
| Tous-Espelosin 2023 |  |  |  |  |  |  |
| Wu 2007 |  |  |  |  |  |  |
| Zhang 2023 |  |  |  |  |  |  |

Domains Judgement

D1: Bias arising from the randomisation process High

D2: Bias due to deviations from the intended interventions Some Concern

D3: Bias due to missing outcome data Low

D4: Bias in measurement of the outcome

D5: Bias in selection of the report result

**Figure S1. Weighted bar plots for distribution of risk of bias assessment within each bias domain for the twenty-six randomised controlled trials.**

**Table S4: Traffic light plot of the domain-level assessment for the five non-randomised studies**

| Paper | D1 | D2 | D3 | D4 | D5 | D6 | D7 | Overall |
| --- | --- | --- | --- | --- | --- | --- | --- | --- |
| Curtis 2016 |  |  |  |  |  |  |  |  |
| Deenik 2019 |  |  |  |  |  |  |  |  |
| Heggelund 2011 | ? |  |  |  |  |  |  |  |
| Magni 2017 | ? |  | ? | ? |  |  |  |  |
| Melamed 2008 |  |  |  |  |  |  |  |  |

Domains Judgement

D1: Bias due to confounding Serious

D2: Bias due to selection of participants Moderate

D3: Bias in classification of interventions Low

D4: Bias due to deviations from intended interventions ? No information

D5: Bias due to missing outcome data

D6: Bias in measurement of the outcome

D7: Bias in selection of the report result

**Figure S2: Weighted bar plots for distribution of risk of bias assessment within each bias domain for the five non-randomised studies.**

**Table S5: Assessment of evidence using Grading of Recommendations Assessment, Development and Evaluation (GRADE)**

| Outcome | Risk of bias (1) | Inconsistency (2) | Indirectness (3) | Imprecision (4) | GRADE | Interpretation |
| --- | --- | --- | --- | --- | --- | --- |
| Body weight,  Composition | | | | | | |
| BMI (kg/m^2^) | Low | Low | Moderate | Low | Low | It is uncertain whether combined nutrition and exercise or stand-alone nutrition or exercise interventions change BMI in people with severe mental illness. |
| Waist circumference (cm) | Low | Low | Moderate | Low | Low | It is uncertain whether combined nutrition and exercise or stand-alone nutrition or exercise interventions change waist circumference in people with severe mental illness. |
| Weight (kg) | Low | Low | Moderate | Low | Low | It is uncertain whether combined nutrition and exercise or stand-alone nutrition or exercise interventions change weight in people with severe mental illness. |
| Blood pressure | | | | | | |
| Blood pressure diastolic (mmHg) | Low | Low | Low | Low | Low | It is uncertain whether combined nutrition and exercise or stand-alone nutrition or exercise interventions change blood pressure diastolic in people with severe mental illness. |
| Blood pressure systolic (mmHg) | Low | Low | Low | Low | Low | It is uncertain whether combined nutrition and exercise or stand-alone nutrition or exercise interventions change blood pressure systolic in people with severe mental illness. |
| Blood |  |  |  |  |  |  |
| Glucose | Low | Low | Low | Low (5) | Low | It is uncertain whether combined nutrition and exercise or stand-alone nutrition or exercise interventions change Glucose in people with severe mental illness. |
| HDL-cholesterol (mmol/L) | Low | Low | Low | Low | Low | It is uncertain whether combined nutrition and exercise or stand-alone nutrition or exercise interventions change HDL-cholesterol in people with severe mental illness. |
| LDL-cholesterol (mmol/L) | Low | Low | Low | Low (5) | Low | It is uncertain whether combined nutrition and exercise or stand-alone nutrition or exercise interventions change LDL-cholesterol in people with severe mental illness |
| Total Cholesterol (mmol/L) | Low | Low | Low | Low (5) | Low | It is uncertain whether combined nutrition and exercise or stand-alone nutrition or exercise interventions change Total cholesterol in people with severe mental illness |
| Triglycerides (mmol/L) | Low | Low | Low | Low | Low | It is uncertain whether combined nutrition and exercise or stand-alone nutrition or exercise interventions change Triglycerides in people with severe mental illness |

BMI: body mass index; HbA1c: HDL: high density lipoprotein; LDL: low density lipoprotein.

(1) Downgraded for high risk of bias as assessed by ROB2, ROBINS-I

(2) Downgraded for inconsistency: heterogeneity (I2) (downgraded further if high heterogeneity), intervention type and duration

(3) Downgraded as some studies were not designed to assess this outcome as a primary outcome (downgraded further if the majority of studies were not designed to assess this outcome)

(4) Downgraded as some studies had a small sample size and hence wide confidence intervals, dietary intervention design and adherence varied introducing imprecision

(5) Downgraded for imprecision due to small number of studies/ fewer participants.

**Meta-analysis**

**Figure S3: Forest plots for trials that assessed the impact of combined interventions on primary outcomes**.

a. Cholesterol
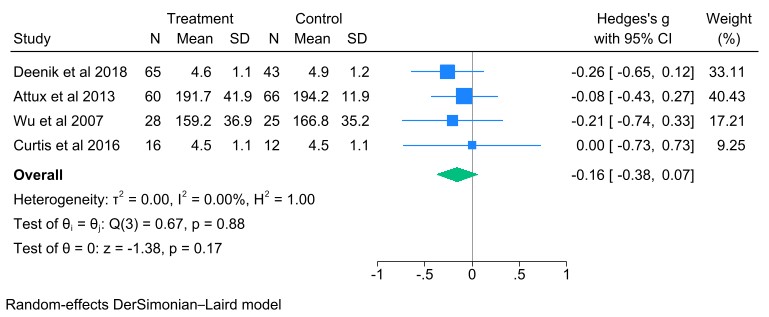


b. Diastolic Blood Pressure
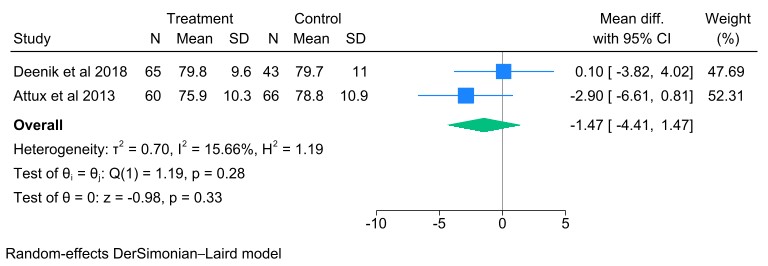


c. Systolic Blood Pressure


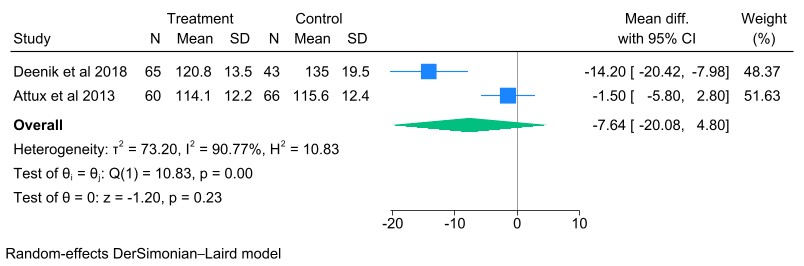


d. Glucose
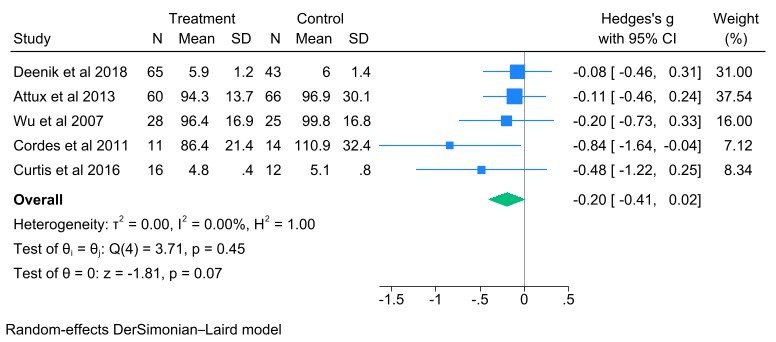


e. High Density Lipoprotein Cholesterol
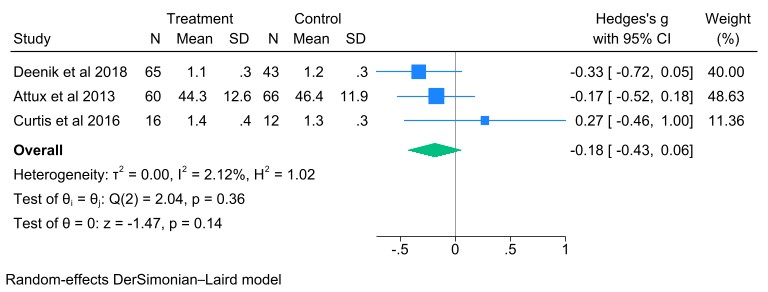


f. Triglycerides


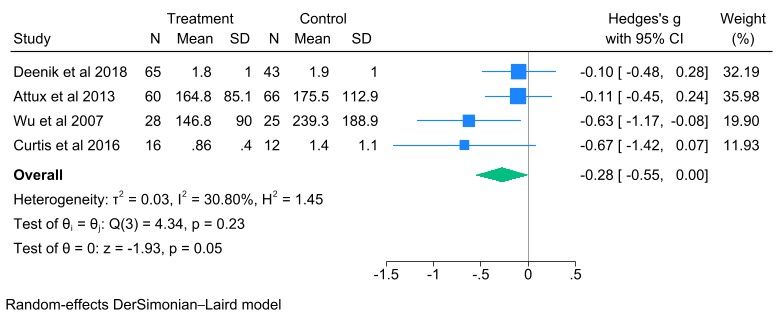


**Figure S4: Forest plots for trials that assessed the impact of nutrition interventions on primary outcomes.**

a. BMI
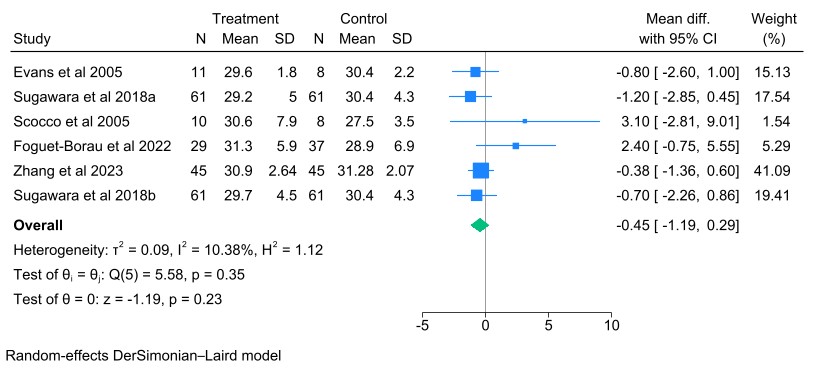


b. Weight


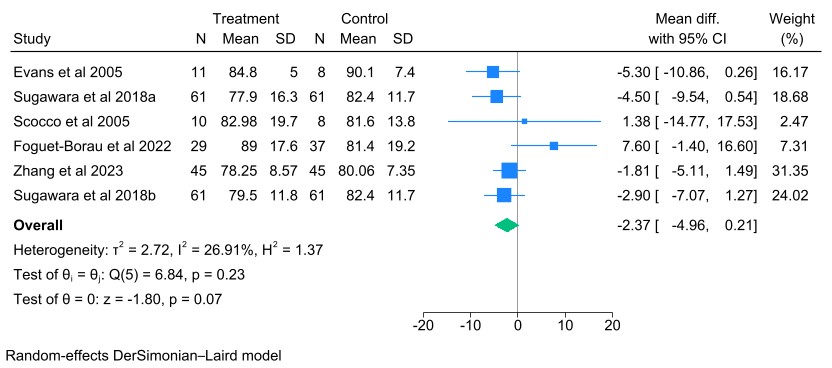


c. Waist


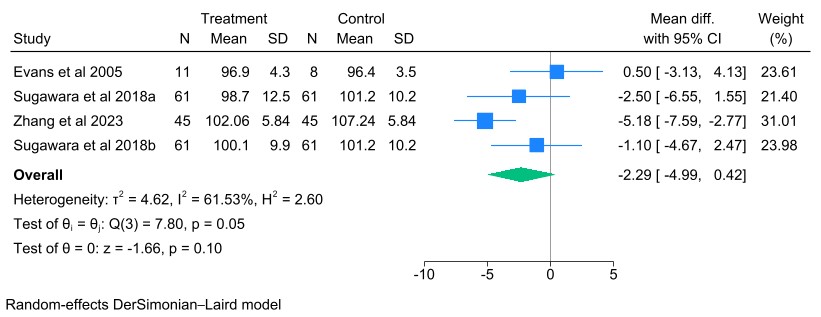


d. Systolic Blood Pressure
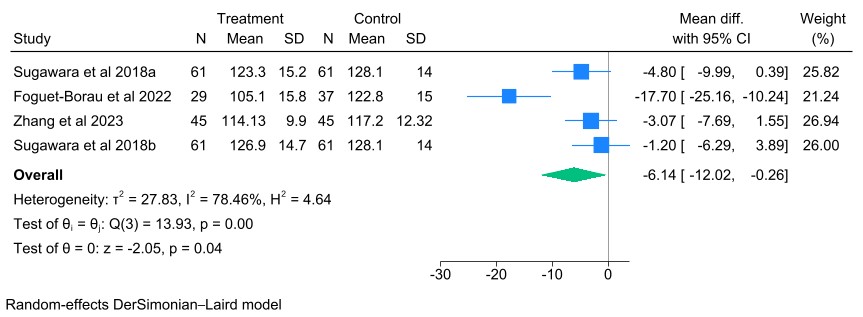


e. Diastolic Blood Pressure


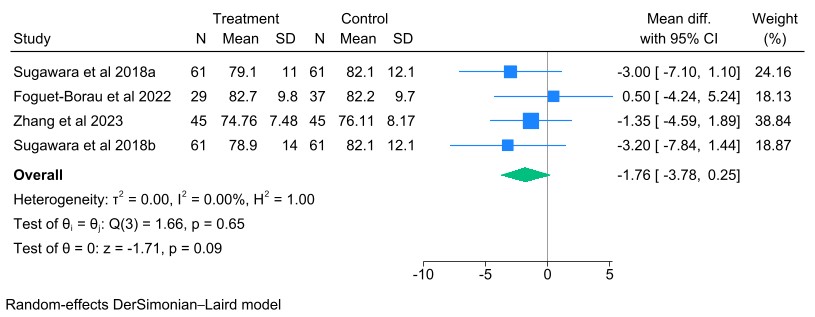


**Figure S5: Forest plots for trials that assessed the impact of exercise interventions on primary outcomes.**

a. Weight


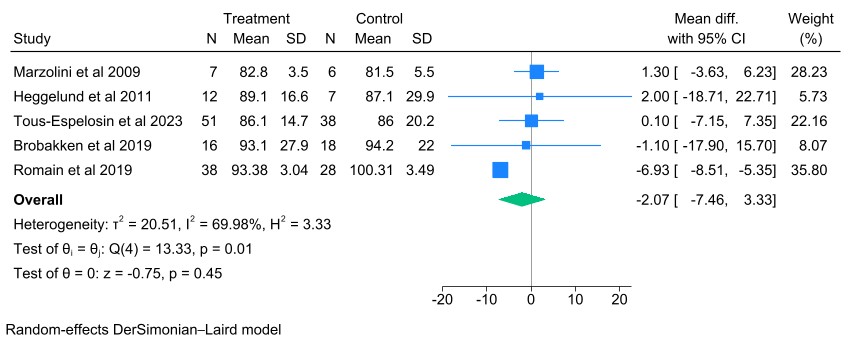


b. Triglycerides


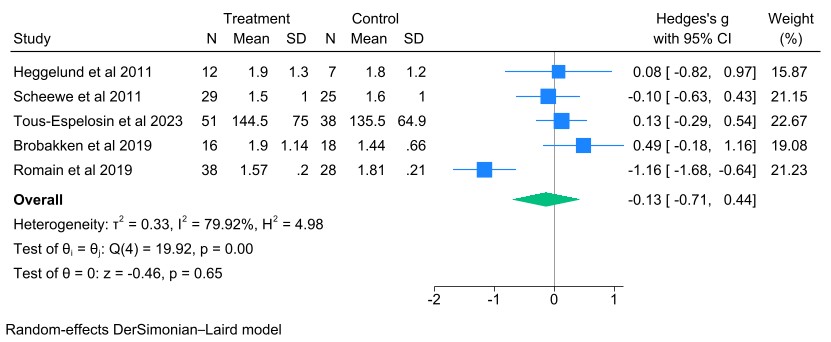


**Figure S6: Funnel plot of the meta-analysis of the included studies that investigated the impact on body weight.**


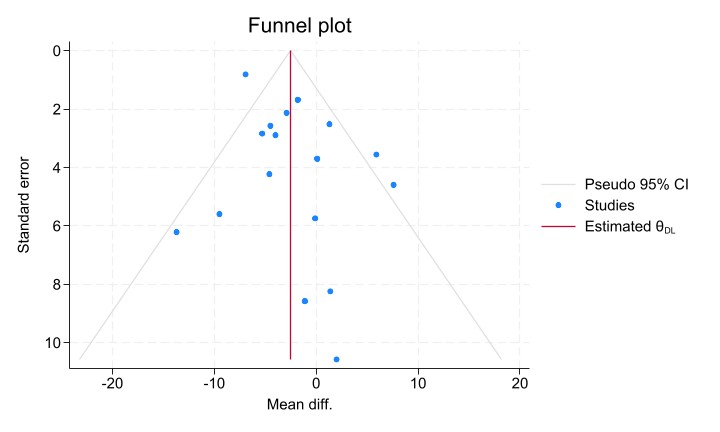

Supplement: sj-docx-1-anp-10.1177_00048674251384054 – Supplemental material for Effectiveness of specialist-delivered interventions in severe mental illness: A systematic review and meta-analysis [file sj-docx-1-anp-10.1177_00048674251384054.docx]
